# Supplementary material for: Diminished Pathogen and Enhanced Endophyte Colonization upon CoInoculation of Endophytic and Pathogenic Fusarium Strains
Source: Microorganisms. 2020 Apr 9;8(4):544. doi: 10.3390/microorganisms8040544 (PMC7232452; doi:10.3390/microorganisms8040544)
Supplement: Supplementary file 1 [file microorganisms-08-00544-s001.pdf]

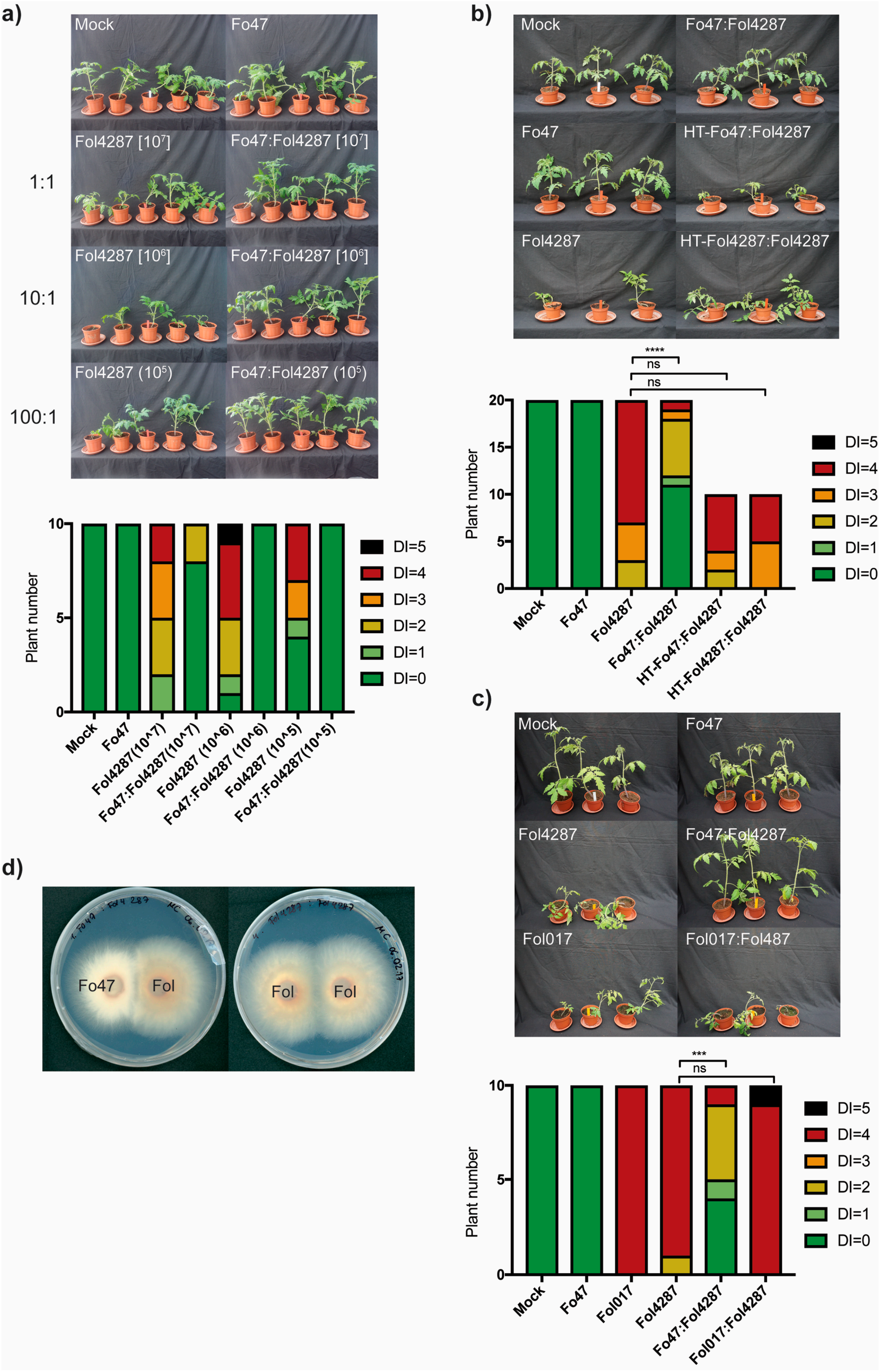
a)
Mock
Fo47
Fol4287 [10⁷]
Fo47:Fol4287 [10⁷]
1:1
Fol4287 [10⁶]
Fo47:Fol4287 [10⁶]
10:1
Fol4287 (10⁵)
Fo47:Fol4287 (10⁵)
100:1
Plant number
0
5
10
Mock
Fo47
Fol4287(10^7)
Fo47:Fol4287(10^7)
Fol4287 (10^6)
Fo47:Fol4287 (10^6)
Fol4287 (10^5)
Fo47:Fol4287(10^5)
DI=5
DI=4
DI=3
DI=2
DI=1
DI=0
b)
Mock
Fo47:Fol4287
Fo47
HT-Fo47:Fol4287
Fol4287
HT-Fol4287:Fol4287
****
ns
ns
Plant number
0
5
10
15
20
Mock
Fo47
Fol4287
Fo47:Fol4287
HT-Fo47:Fol4287
HT-Fol4287:Fol4287
DI=5
DI=4
DI=3
DI=2
DI=1
DI=0
c)
Mock
Fo47
Fol4287
Fo47:Fol4287
Fol017
Fol017:Fol487
***
ns
Plant number
0
5
10
Mock
Fo47
Fol017
Fol4287
Fo47:Fol4287
Fol017:Fol4287
DI=5
DI=4
DI=3
DI=2
DI=1
DI=0
d)
Fo47
Fol
Fol
Fol

Figure S1. Endophyte-mediated resistance set-up. **(a)** Ten-days old tomato seedlings cultivar Money Maker were inoculated with water (mock), Fo47 (10^7 spores/mL), Fol4287 (10^7, 10^6, 10^5 spores/mL) or co-inoculated with Fo47 and Fol4287 (ratio 1:1, ratio 10:1 or ratio 100:1) This experiment was performed twice with similar results; **(b)** Ten-days C32 old tomato seedlings were inoculated with water (mock), Fo47 (10^7 spores/mL), Fol4287 (10^7 spores/mL), or co-inoculated with spores (Fo47 or Fol4287) heat-treated at 60°C for 15 minutes together with Fol4287 (10^7 spores/mL). Disease development was assessed by measuring vascular browning three weeks after inoculation. Disease index (DI) =0 no brown vessels; DI=1 brown vessel(s) only at basal level; DI=2 one or two brown vessel at cotyledon level; DI=3 three brown vessels at cotyledon level, DI=4 all vessesl are brown, DI=5 dead plant. Data was analysed by a non-parametric Mann-Whitney U-test (^{ns}P>0.05; ****P<0.0001); **(c)** Ten-days old C32 tomato seedlings were inoculated with water (mock), Fo47 (10^7 spores/ml), Fol4287 (10^7 spores/ml), Fol017 (10^7 spores/ml), co-inoculated with Fo47 and Fol4287 or Fol4287 and Fol017. Disease symptoms were assessed three weeks after inoculation. Data were analysed by a non-parametric Mann-Whitney U-test (^{ns}P>0.05; ***P<0.001); **(d)** Plugs of seven days old (Fo47, Fol4287) mycelia were placed three cm apart on PDA plates. Pictures were taken after six days. Abreviation: DI= disease index; HT: heat treated.

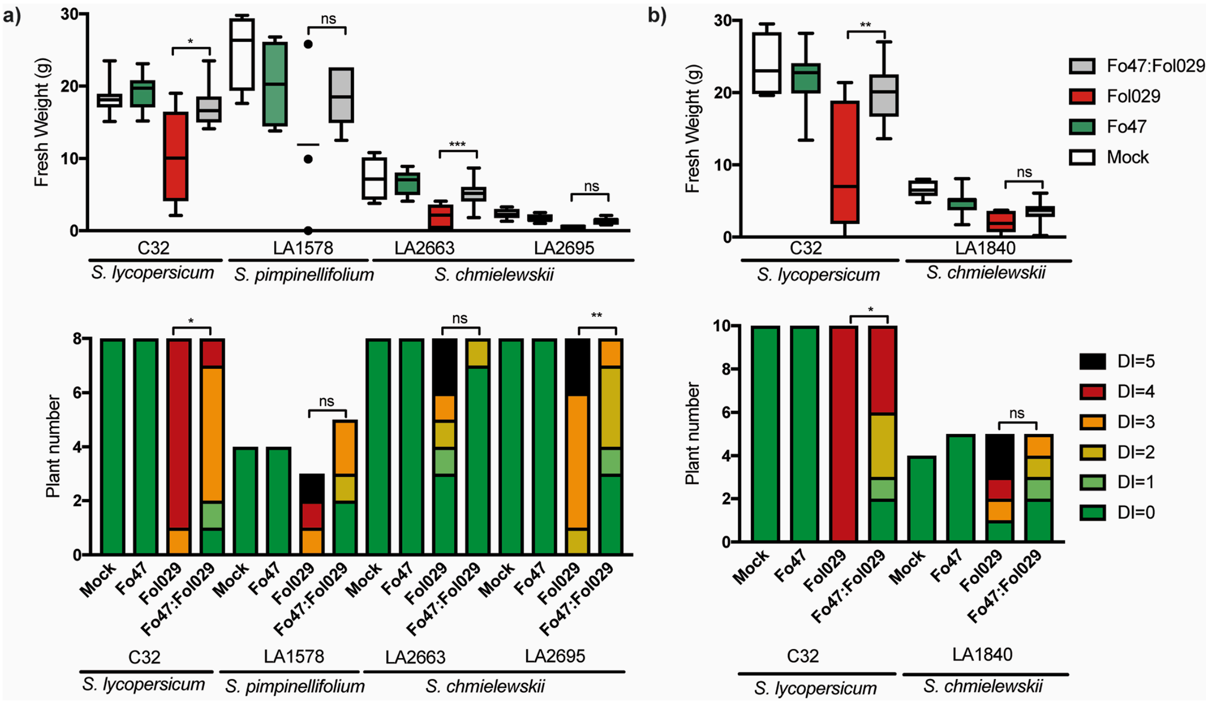

Figure S2. Fo47 can trigger resistance against Fol029 in *Solanum lycopersicum* (C-32), **(a)** *S. pimpinellifolium* (LA1578) and *S. chmielewski* (LA2663, LA2695) and in **(b)** *S. chmielewski* (LA1840). Thirteen days old tomato seedlings were inoculated with water (mock), Fo47, Fol029 or co-inoculated with Fo47 and Fol029. Fresh weight and disease development were assessed three weeks after inoculation. Disease index (DI) =0 no brown vessels; DI=1 brown vessel(s) only at basal level; DI=2 one or two brown vessel at cotyledon level; DI=3 three brown vessels at cotyledon level, DI=4 all vessesl are brown, DI=5 the plant is dead.Data were analysed by a non-parametric Mann-Whitney U-test (^{ns}P>0.05; *P<0.05, **P<0.01; ***P<0.001).

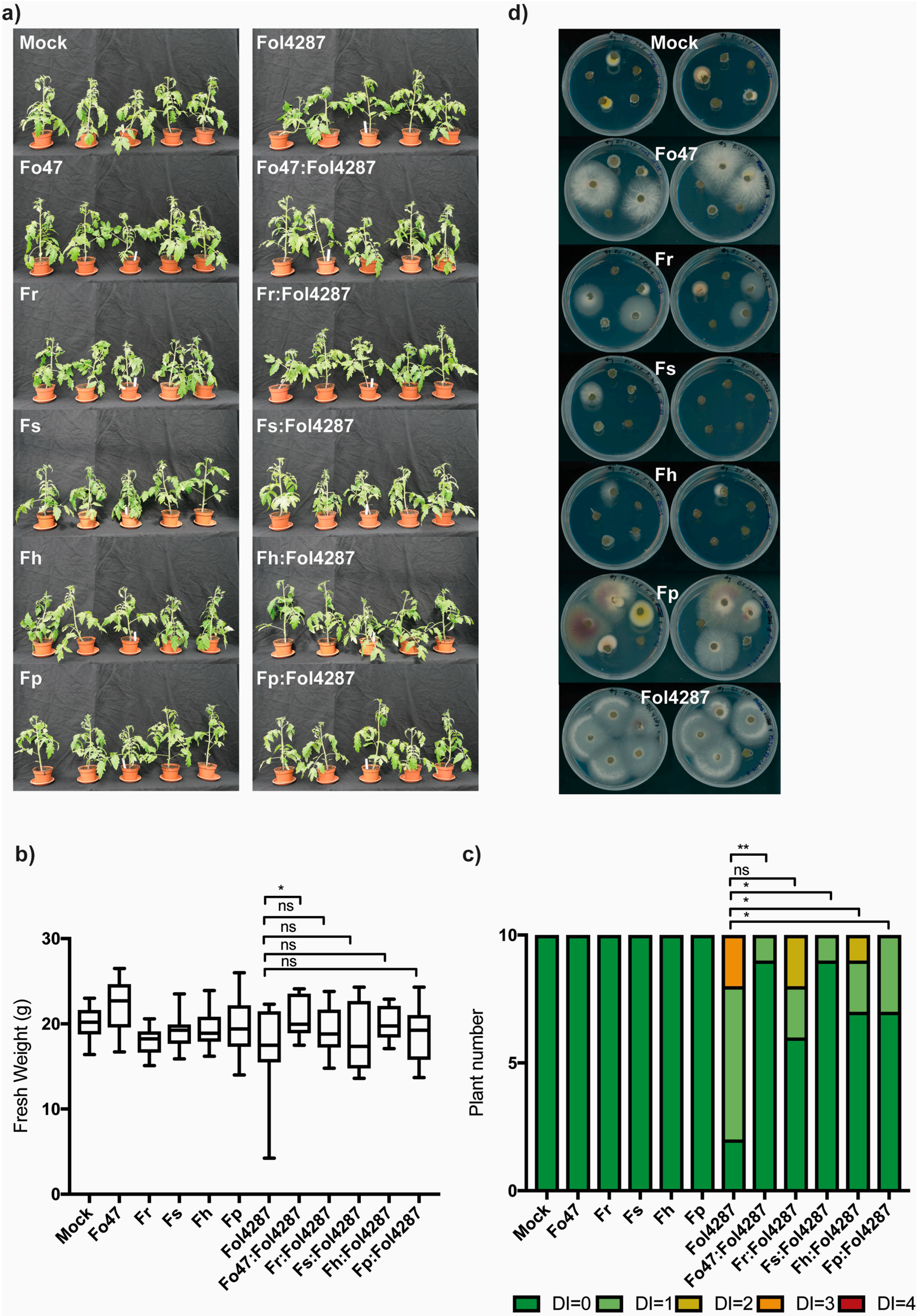

Figure S3. Fo47, *Fusarium redolens* (Fr), *Fusarium solani* (Fs), *Fusarium hostae* (Fh) and *Fusarium proliferatum* (Fp) can suppress *Fusarium* wilt disease in tomato**.** **(a)** Tomato plants three weeks after inoculation; **(b)** Fresh weight and **(c)** disease index of tomato plants three-weeks after inoculation. Disease index (DI) =0 no brown vessels; DI=1 brown

vessel(s) only at basal level; DI=2 one or two brown vessel at cotyledon level; DI=3 three brown vessels at cotyledon level, DI=4 all vessesl are brown, DI=5 the plant is dead Data were analysed by a non-parametric Mann-Whitney U-test ($^{ns}P>0.05$; $*P<0.05$; $**P<0.01$; $***P<0.001$). **(d)** Ten tomato stems pieces from crown level showing *Fusarium* outgrowth on PDA plates after being incubated in the dark for four days in dark at 25°C.

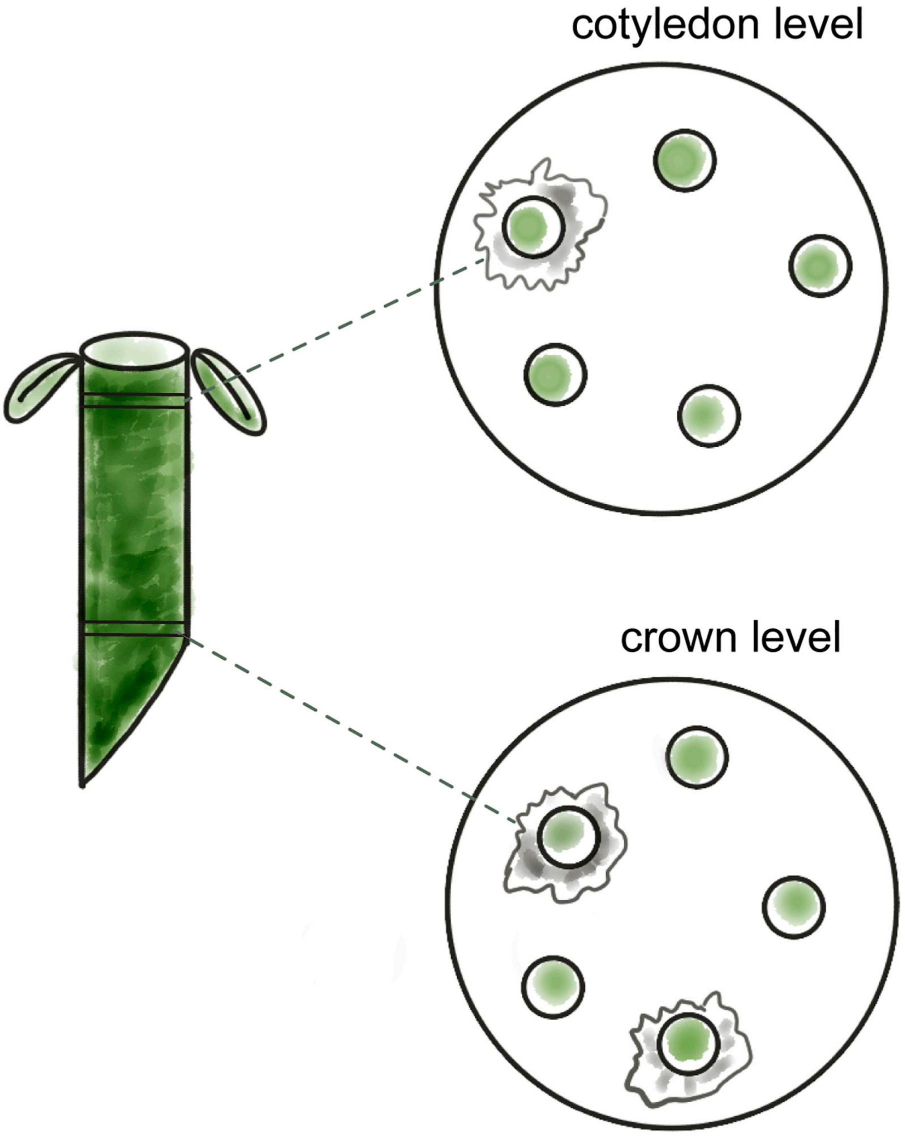

Figure S4. Schematic representation of a tomato cotyledon harvested three-weeks after inoculation, surfaced sterilized with 70% ethanol, washed with sterile water twice. A piece at the cotyledon level and crown level was placed on PDA plates (together with four other tomato stem pieces) and incubated for four days at 25°C in the dark.

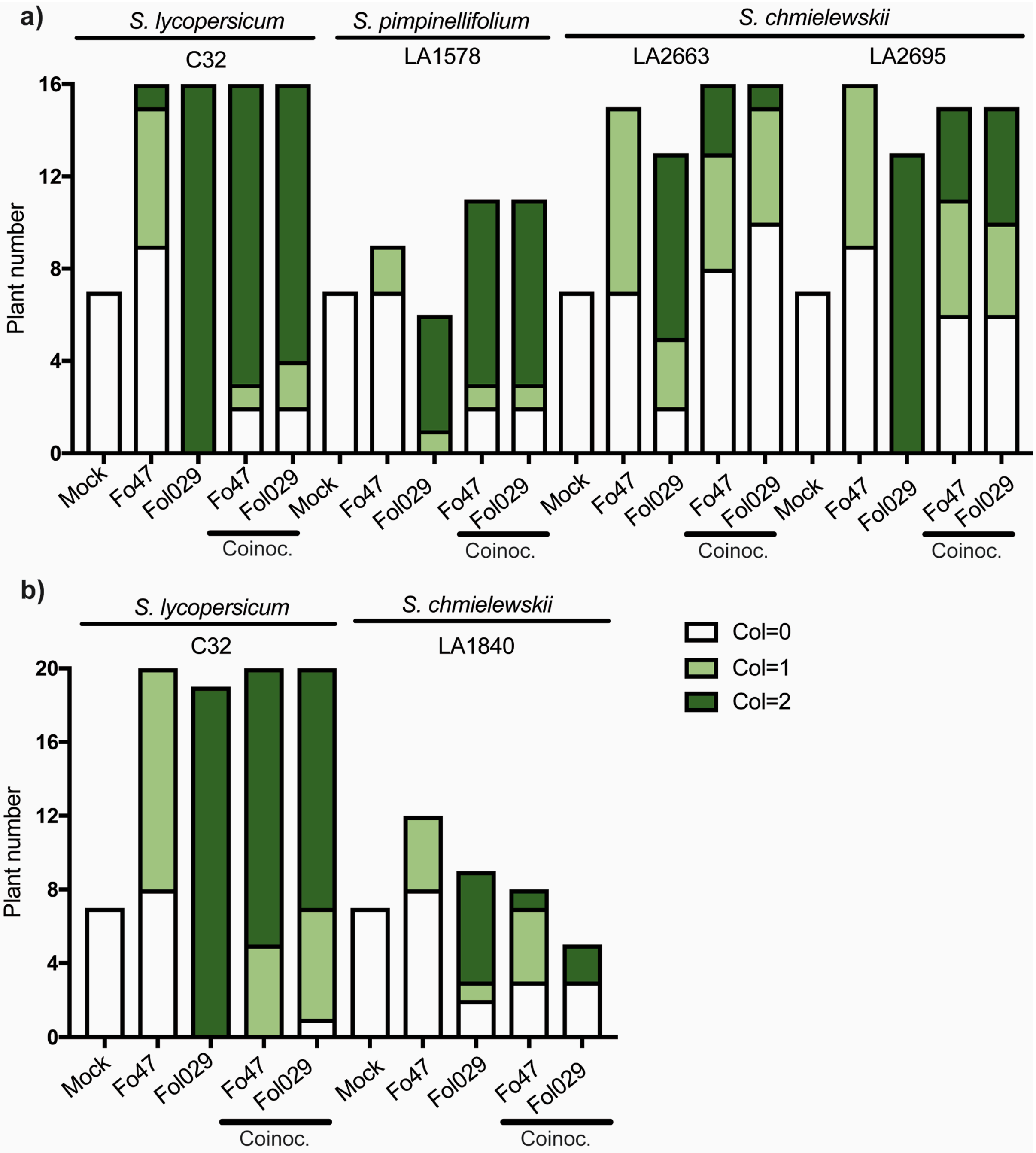

Figure S5. Fo47 reaches cotyledon levels upon co-inoculation with Fol029, while pathogen colonization is reduced in *S. lycopersicum* (C-32) **(a)** *S. pimpinellifolium* (LA1578) and *S. chmielew*ski (LA2663, LA2695); **(b)** *S. chmielewski* (LA1840). Col=0: no Fo outgrowth observed; Col=1: Fo outgrowth at either crown or cotyledon level; Col=2: Fo outgrowth at both crown and cotyledon level. Data represented here combine two independent experiments. Abbreviations: Col= colonization; Coinoc.= co-inoculation.

Table S1. Primer sequences used for (q)PCR analysis.

Gene	Primer names	Forward primer (5’-3’)	Reverse primer (5’-3’)	Annealing temperature	Reference
EF1-alpha	FP889, FP1416	TCGTCGTCATCGGCCACGTC	GGAAGTACCAGTGATCATGTT	55°C	[25]
Tubulin	FP2147, FP2148	CAGTGAAACTGGAGCTGGAA	TATAGTGGCCACGAGCAAAG	60°C	__
IGS	FP8498, FP8499	TTTGCCATACTATTGAATTTTGC	ACTTTTACCTACCCGGCAGCTC	60°C	__
SCAR	FP7069, FP7070	CCTCAACTTCTGATTTAAATATGA	GAGCGAACAACTACAATAAAAG	60°C	[26]
SIX8	FP6994, FP7569	GTATGTCTGATTCTCATGAATAC	GTTATGCAGGCGAGTAAAATG	60°C	__
